# Development of self-help groups for caregivers of children with disabilities in Kilifi, Kenya: Process evaluation

**DOI:** 10.4102/ajod.v9i0.650

**Published:** 2020-07-22

**Authors:** Joseph K. Gona, Charles Newton, Sally Hartley, Karen Bunning

**Affiliations:** 1 Kuhenza for the Children Foundation, Malindi, Kenya; 2 Centre for Geographic Research, Coast, Kenya Medical Research Institute, Kilifi, Kenya; 3 Department of Psychiatry, Oxford University, Oxford, United Kingdom; 4 Department of Psychology, University of Sydney, Sydney, Australia; 5 School of Health Sciences, University of East Anglia, Norfolk, United Kingdom

**Keywords:** caregivers, children with disabilities, community-based inclusive development, self-help groups

## Abstract

**Background:**

Caring for a child with disabilities in a resource-poor setting brings many challenges to the caregiver. We examined the development of self-help groups for caregivers in a rural part of Kenya.

**Objectives:**

To conduct a process evaluation on the development of self-help groups during a 10-month set-up period, focusing on implementation and mechanisms associated with their functional status.

**Methods:**

Using a realist evaluation design, we set up 20 self-help groups for 254 caregivers. An evaluation was conducted to investigate implementation and mechanisms of impact. Implementation focused on caregiver registration, community group support and monitoring visit compliance. Data were collected from group registers, records of meetings and field notes. Mechanisms of impact employed a framework of strengths–weaknesses–opportunities–threats to review the groups at the end of the 10-month set-up period.

**Results:**

Recruitment resulted in registration of 254 participants to 18 groups – two groups disbanded early. Post-evaluation included 11 active and 7 inactive groups. Compliance with the monitoring visits was consistent across the active groups. All groups engaged in ‘merry-go-round’ activities. The active groups were characterised by strong leadership and at least one successful income generation project; the inactive had inconsistent leadership and had dishonest behaviour both within the group and/or externally in the community. Mediators associated with functional status included the following: available literacy and numeracy skills, regular meetings with consistent attendance by the members, viable income generating projects, geographical proximity of membership and strong leadership for managing threats.

**Conclusion:**

Self-help groups have the potential to progress in resource-poor settings. However, critical to group progression are literacy and numeracy skills amongst the members, their geographical proximity, regular meetings of the group, viable income generating projects and strong leadership.

## Introduction

Children represent approximately 50% of the general population, 5% of whom are estimated to have a disability (World Health Organisation [WHO] 2011). More recently, about 95% of 52.9 million children below 5 years with developmental disabilities were estimated to reside in low- and middle-income countries (LMICs) (Olusanya 2018). Compared to 1990 estimates, the authors concluded that there was a lack of significant improvement to the burden of developmental disabilities. The basic needs of the child growing up with a disability, such as shelter, nutrition, clothing, education, health and emotional well-being, are catered for by the caregiver, usually the mother. In LMICs, paucity of information concerning the causes of disability, for example, Kenya (Bunning et al. 2017), limited support services and poor access at community level, makes the caregiver’s role both challenging and onerous (Gona et al. 2018).

Wide variation in rehabilitation services has been reported across the African continent, including poor coordination of delivery, restricted access to services at community level and a continuing need for development work (WHO 2011). Health-based rehabilitation services that exist tend to be clustered around urban-based institutions with reports of serious limitations in coverage and capacity (Njelesani, Couto & Cameron 2011; Parnes, Cameron & Christie 2009).

In the circumstance of limited resources (Mitra, Posara & Vick 2011; Peters et al. 2018) and social isolation, the caregiver and the child with a disability are disenfranchised and potentially marginalised in their own community (Ambikile & Outwater 2012; Bunning et al. 2017; Trani et al. 2011). The family’s finances are impacted by the extra expenses associated with meeting the child’s needs (Ambikile & Outwater 2012; Gona et al. 2016). A report from Sierra Leone found that families with persons with severe disabilities spent on average 1.3 times more on healthcare than families where disability was not present (Trani et al. 2011).

These challenges are compounded by a lack of information about the causes of disability and competing explanations in the community based on cultural superstitions and negative images, for example, the child’s disability is attributed to curses or evil spirits (Bunning et al. 2017). Furthermore, with an estimated third of youth (12–14 years) and approximately 60% of those between the ages of 15 and 17 years not attending school in sub-Saharan Africa (www.uis.unesco.org/en/topic/education-africa), it is likely that caregivers will lack skills of literacy and numeracy to help advance their quality of life.

Not surprisingly, long-term caregiving in low-income countries has been associated with fatigue and parenting distress (Gona et al. 2014). Furthermore, children with disabilities are more likely to have lower school attendance than their non-disabled counterparts with limited support available generally. Local access to rehabilitation services is cited as a right by the United Nations Convention on the Rights of People with Disabilities (UN 2006), although the reality faced by most people in low-income countries is one of scarce and frequently inaccessible resources.

Community-based inclusive development (CBID), formerly community-based rehabilitation (CBR), provides the potential to circumvent existing gaps in available rehabilitation support. Initiatives based on the WHO CBR Matrix (2010) continue to evolve and grow in more than 90 countries worldwide, focusing on strategies for ‘rehabilitation, equalisation of opportunities, poverty reduction and social inclusion of people with disabilities’.

However, published studies have been criticised for the lack of research rigour (Finkenflugel, Wolffers & Huijsman 2005). Iemmi et al.’s (2015) systematic review identified modest benefits for people with mental disabilities and their caregivers whilst also acknowledging ‘methodological constraints’ (p. 6) in the cited studies. ‘Empowerment’ is one of the five domains of the WHO matrix (WHO 2010), the others being ‘livelihood’, ‘education’, ‘health’ and ‘social’. Seminal studies by Kieffer (1984) and Zimmerman and Rappaport (1988) supported the idea that psychological empowerment includes personal control, a sense of competence, a critical awareness of the sociopolitical environment and participation in community organisations and activities.

Zimmerman and colleagues (e.g. Perkins & Zimmerman 1995; Zimmerman & Rappaport 1988; Zimmerman & Warschausky 1998) identified three key domains: *intrapersonal*, how people think about their capacity to influence change utilising critical understanding of context; *interactional*, how people contribute to transactions with other people and the environment; and *behavioural*, how people act to influence change in the surrounding environment, for example, through participation in community organisations and activities. The latter point is relevant to CBID initiatives such as ‘self-help groups’ (SHGs), which are identified in the ‘empowerment domain’ (WHO 2010) and bring new opportunities for social connections and support in the community.

Self-help groups are grassroot-level organisations that build on the traditions of collective savings and shared livelihood activities. Their purpose is to promote peer assistance and cooperation for the mutual benefits of the members (Gugerty, Biscaye & Anderson 2019). A variety of models have been used in LMICs for different purposes, including education for an alternative livelihood in Kenya (UNESCO 2015); promoting well-being amongst people with mental health needs in Ghana (Cohen et al. 2012); raising awareness of disability issues in the community in South Africa (Adams & Galvaan 2016); social support through training for caregivers of children with disabilities in Ghana (Zuurmond et al. 2019); promoting agricultural practices across remote, rural regions of sub-Saharan Africa (Self-help Africa) and Asia (Atteraya, Gnawali & Palley 2016); and use of microfinance to mitigate the effects of humanitarian crises in Ethiopia (Tearfund 2017). Few formal evaluations that distinguish independent variables (e.g. interventions) from co-variables (e.g. environmental factors) have been reported (Gugerty et al. 2019), with the exception of studies originating in Asia. Atteraya et al. (2016) found that individual capabilities (e.g. educational experience, home assets, autonomous decision-making) were significantly correlated with active participation in the SHGs. This finds resonances in Patil and Kokate’s (2017) analysis of factors underpinning participant attitude formation towards SHGs that included ‘coping ability’, ‘personality traits’, ‘resource utilisation and building’, ‘entrepreneurial attributes’, ‘organisational governance’, ‘financial inclusion’ and ‘economic upliftment’. Another study in India considered group process characteristics such as commitment and cooperation of members, absence of conflicts and transparency of communication to be critical to positive SHG outcomes (Govindarajan & Padhmanabhan 2013).

Regarding impacts, reported outcomes associated with SHG participation include the following: more positive attitudes and a reduction in perceived isolation (Zuurmond et al. 2019), and improved financial and social support (Cohen et al. 2012; Swain & Wallentin 2012). A systematic review of SHGs for women in Asia, sub-Saharan Africa and the Caribbean revealed economic gains and political empowerment (Brody et al. 2017).

Reported challenges to SHG participation include the following: competing priorities and time poverty, managing the tensions between individual and group goals (Adams & Galvaan 2010); differences in community status (e.g. caste differences in India); disappointment in expected benefits; and stigma associated with membership (Brody et al. 2017). However, inadequate documentation of group processes remains a problem in attributing change to any one model of self-help.

To understand the functional status of the SHGs at the end of a 10-month set-up period, the current study aimed to carry out a process evaluation. The research question was: What characteristics and processes define the functional status (active vs. inactive) of SHGs?

## Research methods and design

The project adopted a realist evaluation design (Pawson & Tilley 1997), which recognises that programmes work in different ways for different people. It was expected that the development of 20 SHGs in different geographical locations would be influenced by the experiences, beliefs and attitudes of the participants; the available opportunities; access to resources relevant to the context; and environmental conditions.

## Setting and sample

The setting was Kilifi County (area: 12 610 km^2^; poverty level: 71.4% – Kenya Commission Revenue Allocation). The sample was composed of caregivers of children with disabilities across 10 sub-locations in Kilifi County. The primary caregiver was included if:

- she or he was 18 years old and above and cares for a child (0–15 years) with a developmental disability present from birth, noted in first 5 years of life or considered long-term
- parental report identified the child as showing a deficit(s) in one or a combination of the following areas: seeing, hearing, moving, dribbling, drinking and eating, paying attention, sitting still, learning, understanding, or experiences epileptic seizures (fits) (derived from the first section of the Communication Disability Profile: Baker & Hartley 1999)
- the child’s disability was associated with a primary condition, for example, cognitive impairment, deafness, visual impairment, autistic spectrum condition, cerebral palsy and multiple disabilities.

Caregivers were excluded where the child’s condition was temporary and possibly associated with a medical trauma, for example, fractured limb, and likely to resolve with appropriate treatment, or related to a need that could be resolved through the provision of corrective devices, for example, glasses for myopia.

**FIGURE 1:**
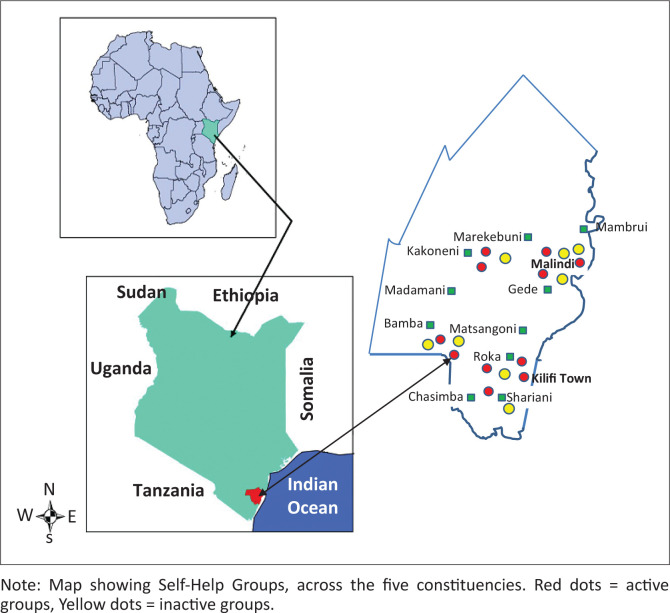
Map of Kilifi County showing the locations of the self-help groups across the five constituencies: Functional status indicated.

To engage the community in each targeted sub-location and to secure the support of the sub-chief responsible for community affairs, a field worker, a resident of Kilifi, went to the designated sub-chief’s office to arrange a visit by the project co-ordinator (also a local resident). At the meeting, project information was provided to the sub-chief and any questions were fielded. Caregiver recruitment was carried out by 20 existing community groups (women groups [WG]; community health worker groups [CHW]), who had participated in a previous study on disability awareness training (Gona et al. 2018). Each of two groups per sub-location was asked to identify around 15 caregivers of children with disabilities who were known to them, making a target recruitment number of 300 caregivers (see Figure 1 for the location of the SHGs across Kilifi County). An inaugural meeting was arranged for each SHG development site. Members of the WGs and CHW groups who had identified caregivers in their own communities accompanied the caregivers to this first meeting to learn about the project. Informed consent was recorded for those caregivers who wanted to participate in the development of SHGs, whereby information was read out, questions were addressed and participation decisions were recorded by signature or thumbprint.

## Process evaluation

Process evaluation was carried out during the set-up phase of the project (over 10 months). It focused on two domains (Moore et al. 2015): (1) implementation, or the approaches taken to set up and support the SHGs with a focus on group set-up (caregiver mobilisation and registration, monitoring visits completed and adaptations); and (2) mechanisms of impact, or group responses to the development process, with a focus on group activities and membership, processes and characteristics (internal or external to the group) and their association to group functional status.

After the initial meeting for information sharing and recording consent, those who were interested in taking part were registered individually using a prepared Excel spreadsheet addressing the following fields: sex, age range, marital status, educational level, number of children with and without disabilities and quality of life indicators (quality of dwelling, caregiver clothing and footwear, number of meals served and livestock owned). Each group was encouraged to assign the roles of chairperson, treasurer and secretary amongst the membership, to agree on a name and to start up an income generating activity to increase their available resources. Each group was given a hard-backed exercise book and pen to record their meetings (date and time; members present; items discussed; income). Post-registration, monitoring visits were carried out by the project co-ordinator, who as a resident of Kilifi was familiar with the culture and conversant in all the local languages. Each group was visited at once monthly intervals, arranged in advance by mobile phone communication with the chairperson. The visits took the form of question–answer interactions with the group, review of their ledger on member attendance and activities – supplemented by an oral report, identification of any difficulties experienced with problem-solving as required. Field notes were recorded *in situ* and later entered into a prepared Excel spreadsheet recording co-ordinator role assignment, group activities and observations made by the researcher.

At the end of the set-up period, a comprehensive review of all the groups was conducted by examining the two domains of (1) implementation and (2) mechanisms of impact. The former (1) focused on caregiver mobilisation by community groups, caregiver registration data, monitoring visits completed and adaptations. The latter (2) focused on the groups’ constitutions, activities, processes and characteristics (internal or external to the groups). To evaluate implementation of the SHG set-up, descriptive statistics were applied to the data (participant demographics, monitoring visit compliance and any adaptations recorded in the field notes) according to group functional status (active vs. inactive). To evaluate the mechanisms of impact, the entire data set was reviewed using a framework of strengths–weaknesses–opportunities–threats (SWOT: see Helms & Nixon 2010), with the first two components addressing factors internal to the group composition, and the latter two addressing external factors. The SWOT analysis was carried out collaboratively by the first author, a native of the area, who was responsible for group facilitation and monitoring visits, and the last author, a visitor to Kilifi, who provided a remote perspective. Each SHG was reviewed in succession and their characteristics recorded on a prepared SWOT matrix initially. This involved review of the registration characteristics of the caregivers, their quality of life indicators, recorded group compliance rates with monitoring visits and field notes from visits (identifying group income generating activities). In addition, the last author asked the first author to describe each group in his own words using prompts such as: How do the members function as a group? What are their particular strengths or weaknesses? What difficulties have the group encountered? The research co-ordinator’s responses were added to the appropriate section of the SWOT matrix.

A second iteration involved the last author reviewing each SHG’s completed SWOT matrix, comparing them for commonalities and differences and making adjustments as required. This was then reviewed with the first author until consensus on the content of each SHG’s SWOT matrix was established. The last stage involved producing two summary SWOT matrices for the active groups and the inactive groups. Similar items were categorised and assigned a label. These were reviewed and discussed by the two researchers until agreement was achieved. Finally, a single SWOT matrix was rendered that combined the two SWOT summary matrices indicating commonalities and differences according to functional status.

## Ethical considerations

This study was approved by the Scientific Ethics and Review Unit (SERU) of Kenya Medical Research Institute (KEMRI) (approval number: SERU 0016/3132) in Nairobi, Kenya.

## Results

### Implementation

The community groups (10 CHW; 9 WG) identified around 280 caregivers out of the targeted 300 to start up SHGs. One WG failed to identify and mobilise any caregivers, which left 19 groups for development, as shown in Figure 1. However, only 18 groups achieved registration of the caregivers because of one group disbanding shortly after mobilisation. Of the remaining 18 SHGs, the functional status at the end of the set-up period was as follows: 11 active groups (operational) and 7 inactive groups (disbanded). Figure 1 shows the location and functional status of the 19 groups. Tables 1 and 2 summarise the characteristics of the registered caregivers according to group functional status post-set-up.

**TABLE 1:** Summary of compliance with implementation across active and inactive groups post-registration.

SHG		Average membership			Monitoring visits				No. of SHGs doing activities	
Functional status: sum	Mobilisation ratio CHW:WG	Members		Male:Female	Complete		Unsuccessful		Merry-go-round	Other
		Median	Range		Median	Range	Median	Range		
Active: 11	6:4	14	5–20	2:13	10	-	0	-	11	7
Inactive: 7	5:2	12	12–20	1:12	3	2–5	3	2–5	7	0

SHG, self-help groups; WG, women groups; CHW, community health worker groups.

**TABLE 2:** Summary of caregiver characteristics registered to 18 self-help groups: functional status indicated.

Variable	Active (*n* = 154)		Inactive (*n* = 100)		Sum (*N* = 254)	
	*n*	%	*n*	%	*N*	%
**Age range, %**						
< 20	2	1	2	2	4	2
21–39	79	51	39	39	118	46
40+	74	48	59	59	133	52
**Educational level, %**						
No formal	68	44	48	48	116	46
Primary – incomplete	36	23	23	23	59	23
Primary – complete	37	24	25	25	62	24
Secondary	13	8	4	4	17	7
**Marital status, %**						
Single	9	6	4	4	13	5
Married	106	69	73	73	179	71
Divorced	16	10	8	8	24	9
Widow	23	15	15	15	38	15
**No. of children at home, %**						
1–2	18	12	17	17	35	14
3–6	90	58	53	53	143	56
7–10	40	26	25	25	65	25
11+	6	4	6	6	12	5
**No. of children with disabilities, %**						
1	146	95	91	91	237	93
2	6	4	8	8	14	6
3	2	1	1	1	3	1

As shown in Table 1, the CHW groups were responsible for bringing caregivers together for 11 SHGs compared to 6 SHGs by the WGs. The amount and frequency of meetings varied across the SHGs. The active groups met at weekly intervals, which amounted to around 40 meetings over 10 months, each meeting lasting 2–3 h.

Monitoring visits were successful according to the monthly arrangements made, as shown in Table 3. Of the inactive groups, two of the seven came together as a group for less than a 3-month period before disbanding. The remaining five groups continued to meet for between 3 and 5 months. Follow-up visits were arranged when the membership failed to attend a monitoring visit, by contacting the relevant chairperson. However, these were largely unsuccessful (see Table 1). The inactive SHGs showed inconsistent attendance and poor representation of the membership at the visits with as few as one or two members being present on occasions.

**TABLE 3:** Summary of demographic characteristics for caregivers registered to 18 self-help groups: functional status indicated.

Variable	Active (*n* = 154)		Inactive (*n* = 100)		Sum (*N* = 254)	
	*n*	%	*n*	%	*N*	%
**Caregiver clothing, %**						
Quality – poor	0	0	0	0	0	0
Quality – good	154	100	100	100	254	100
Footwear – yes	143	93	6	39	149	46
Footwear – no	11	7	94	59	105	52
**State of home dwelling, %**						
Mud & thatch – poor	56	36	35	35	91	36
Mud & thatch – good	29	19	15	15	44	13
Iron roof	37	24	33	33	70	26
Concrete	32	21	17	17	49	19
**Meals served per day, %**						
1	15	10	12	12	27	11
2	55	36	44	44	99	39
3	80	52	42	42	122	48
4	3	2	2	2	5	2
**Livestock, %**						
Chicken	96	62	62	62	158	62
Duck	32	21	21	21	53	21
Goat	68	44	38	38	116	46
Cow	28	18	23	23	51	20
None	39	25	24	24	63	25

All the 18 groups decided on a name and assigned officer roles amongst their membership (chairperson; treasurer; secretary). In some cases, an additional role was assigned – that of a co-ordinator who facilitated the work of the other officers. Typically, this role was fulfilled by a member of the community group who had been involved in the original mobilisation of the caregivers.

### Mechanisms of impact

As shown in Table 1, responses to the development process were initially favourable with all 18 SHGs embarking on merry-go-round activities, where, according to the agreement of the group, each member contributes either a small sum of money (e.g. around Ksh 50) or food stuffs (e.g. bag of maize flour, sugar).

Once the treasurer has collected the member contributions, the collection is divided amongst three to four members who use their allowance to improve the situation at home, for example, cooking cakes to sell at profit. In addition, seven of the SHGs (active) embarked on group income generating projects (other activities), including, for example, making and selling liquid soap, makuti for roofing; rearing livestock (chickens, goats); breaking stones into gravel for building.

There was minimal difference between the active and inactive groups in terms of caregiver characteristics (see Table 2). As shown in Table 2, between 23% and 25% of the members of both active and inactive groups had completed primary education. However, a slightly higher percentage of active group members had attained a secondary level of education (8%) compared to 4% of the inactive group’s membership.

In terms of demographic characteristics, there was again little difference between the groups according to their functional status (see Table 3). Caregiver clothing, specifically footwear, was much more common amongst the active group members (see Table 2). In addition, 56% of the inactive group members served two or less meals per day compared to those in the active groups (46%).

Internal factors were reflected in strengths and weaknesses of all the groups, and external factors in the opportunities and threats. However, the balance varied between active and inactive groups, with the former being weighted towards strengths and opportunities, and the latter towards weaknesses and threats.

**FIGURE 2:**
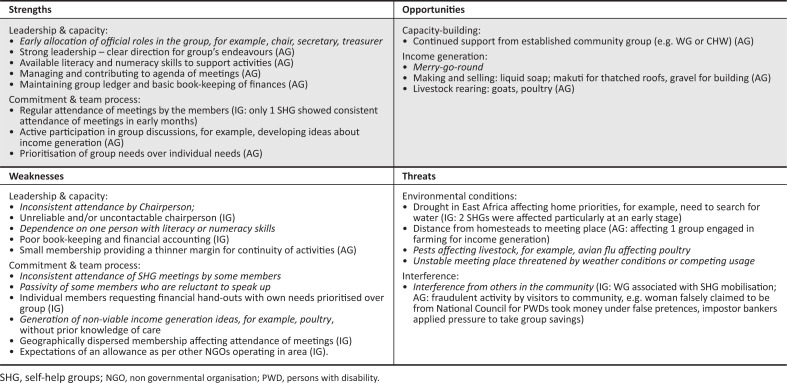
Summary of strengths–weaknesses–opportunities–threats analysis for active and inactive groups: shared characteristics and processes indicated in italics (separate characteristics are indicated in brackets: active groups [AG] and inactive groups [IG]).

As summarised in Figure 2, the majority of the active groups had a strong and consistent leader, often with the continued support of members of the local CHW or WG. Sometimes, a person had dual membership of both the SHG and a community group, which enabled the sharing of group experiences and skills. In one group, a CHW continued as a member of the SHG, supporting the leader by contributing her literacy and numeracy skills for recording group discussions and work transactions. A stable membership, committed to the group’s activities, was evident in the regular attendance of weekly meetings and the commitment of team members. Critical mass appeared to be important to the development capacity of the groups, with activity success being threatened in smaller groups by a lack of persons to input their labour. Early identification and management of threats, for example, breaking away from a large chaotic group, risk management of income generation activities, enabled the groups to grow. However, the active groups were not without weaknesses. Two groups relied on a few caregivers with poor commitment from the rest of the members. Such vulnerabilities demanded close monitoring and support for those members. Restricted literacy and numeracy skills amongst members were a problem in one or two groups, affecting management of group finances and recording decisions. Threats that were identified tended to centre on interference from external people, for example, fraudulent activity by people in the community attempting to take money from the group, individual members demanding hand-outs and environmental conditions such as drought – the latter affecting food and water supplies. Notice of such threats was brought to the attention of the researcher who gave advice that served to mitigate any potentially disastrous impacts on the group. Lack of a secure meeting place was a minor threat for a couple of groups, for example, located under a tree that would be affected by the rainy season; an incomplete building structure because of be completed for use by the police service.

Opportunities for the development of key skills, such as chairing a meeting, recording the notes and the financial transactions, were available to all groups. However, income generation was grasped by the groups with variable success. The seven groups who started up income generation activities were all in the active category.

The inactive groups demonstrated inherent weaknesses from a very early stage, such as poor and inconsistent leadership. This made them vulnerable to external threats, even though similar threats were identified for the active groups. Together with a lack of consistent leadership, a geographically dispersed membership affected group cohesion. In one area particularly (Bamba), the effects of drought threatened the set-up of groups from the start, as caregivers had to prioritise the search for water for their families over attendance of SHG meetings. In some cases, the fraudulent behaviour of others (both within the group and externally) who took money and food stuffs under false pretences affected the morale of members and their motivations to keep going.

## Discussion

Out of 20 targeted SHGs, two groups failed to achieve registration. At the end of the set-up period, seven groups out of the 18 registered groups had disbanded. Eleven groups were still functional. All 18 groups had decided on a named identity, assigned officer roles in their group and embarked on merry-go-round activities. However, membership and compliance with monitoring visits varied across the groups.

Characteristics associated with the collapse of the inactive groups included the following: poor leadership, inconsistent attendance and failure to comply with monitoring visit requirements; poor availability of key skills, such as numeracy and literacy; interference from former members and others outside of the group; and harsh environmental conditions affecting livestock and access to water. In contrast, the active groups appeared to have greater commitment amongst the membership and attendance of meetings, with monitoring visits happening as planned; better access to numeracy and literacy skills amongst the members (with the exception of one SHG); the means for addressing external threats through timely advice; and capacity to develop start-up projects for income generation.

Some groups did not progress beyond initial registration because of competing needs in the home brought about by the extreme drought conditions affecting East Africa. Maslow’s motivation theory based on a hierarchy of need places this at the foundation level: physiological (Koltko-Revera 2006; Maslow 1943). Without water, the threat to family survival was present. Thus, the search for water was prioritised over participation in the SHG development, which is consistent with challenges identified by Adams and Galvaan (2010). In addition, caregiver dispersal over a large geographical area may have been a factor in their coming together for meetings. This was despite a recruitment strategy via established community groups operating in a defined geographical area.

Transport limitations and a lack of finances would also likely have affected their attendance (Ambikile & Outwater 2012; Gona et al. 2016). Beyond a ‘physiological’ level of need, threats to ‘safety’ were present in all the groups, active and inactive. The mere act of registering with an SHG meant identifying themselves as caregivers of a child with disabilities and possibly opening themselves to aversive responses from the community where stigma was present (see Bunning et al. 2017). This may have been a factor in the failed registration of caregivers in one SHG after their initial mobilisation.

Attaining a level of ‘belonging and love’ (Maslow’s third level) could be seen to be dependent on the established ‘safety’ of the group and its members. Individuals asserting their own needs over those of the membership brought tensions to some groups, which resonates Adams and Galvaan (2010) and Brody et al. (2017). However, greater threats were encountered from persons external to the SHG development. Whilst all the groups encountered threats from dishonest individuals in the community, the inactive SHGs experienced such threats very early on – in the first 2–3 months. It is possible that the embryonic status of the groups rendered them as vulnerable to disruption. In contrast, the later threats to the active groups happened at a time when relationships amongst the caregivers had been established. This corresponds to Maslow’s third level of need: ‘love and belonging’. There was commitment to the group processes such as the monitoring visits, which provided opportunities for leveraging help on how to not only address problems faced by the group, but also to progress their activities. Empowerment theory as defined by Kieffer (1984) and Zimmerman and Rappaport (1988) would explain this as the growth of control and awareness of the sociopolitical context in which the groups were functioning. The merry-go-round activities were designed to support trust amongst the members (critical to a sense of belonging), as well as providing learning opportunities for handling goods and money as a precursor the income generation projects. However, the inactive groups faltered at this stage and did not progress to livelihood activities, compared to 7 out of the 11 active SHGs. Nevertheless, these activities were critical components of capacity-building. The members gained important experiences in the handling of goods and money, leading onto income generation projects, which reflects Cohen et al.’s (2012) findings. In this context, attainment of Maslow’s higher levels of ‘esteem’, where recognition of self contributes to developing agency, and ‘self-actualisation’, where aspirational potential and the desire to affect change, was relevant.

The extent to which educational level of achievement amongst the membership was important to group sustainability has relevance. Limited fulfilment of caregiver education was generally consistent with recent statistics in sub-Saharan Africa (www.uis.unesco.org). Furthermore, there was greater representation of caregivers who had completed their secondary education in the active groups compared to the inactive. This difference in the active and inactive group membership is consistent with Atteraya et al. (2017) and Patil and Kokate (2016) who asserted the critical importance of individual capabilities to active participation, which included educational background. It was the case that the majority of the groups, active and inactive, relied on just two or three members with the greatest competence in literacy and numeracy, for organising and recording the group’s activities. Having ties with an already established community group, for example, CHW or WG, either through affiliation or through dual membership of two groups (SHG and CHW/WG), brought essential capabilities and prior experience, which may have had a positive effect on group operations. However, the inactive groups had lower access to someone with secondary-level education generally. Whilst officer roles were assigned in all the groups, leadership was a critical component of the business conducted by the groups. The strong leadership in the active groups, which was always associated with primary or secondary educational level of achievement, supported what Zimmerman and colleagues referred to as a critical understanding of context and how to bring about change (e.g. Zimmerman & Rappaport 1988; Zimmerman & Warschausky 1998). Thus, the leader may have affected the direction taken by the group in terms of income-generating projects.

### Strengths and limitations

In a context of scarce reporting of development work of this nature, the strengths of the current study lie in the report of contrasting features of active and inactive groups. However, information on caregiver attendance of group meetings was recorded inconsistently and could not therefore be reported with any accuracy. The SWOT analyses were conducted at the end of the set-up period. However, a SWOT analysis at the midway point may have yielded further information about the development process. This would require more extensive resources for the research.

## Conclusions and implications

The functional status of SHGs, their active or inactive status at the end of a set-up period, appeared to be associated with characteristics and processes both within and external to the groups. Threats to the new enterprise of SHGs were present for all the groups. Motivations to participate in the groups were undoubtedly affected by drought, particularly for the communities worst affected by the environmental conditions. Beyond competing physiological needs, the timing of threats in the group’s development process seemed to be important. Early disruptions rendered some groups vulnerable to dissolution. Of course, the monitoring visits provided opportunities for leveraging help once a sense of trust and belonging in the group had been established. Thus, compliance with monitoring visits appeared to be critical to group survival and growth in the set-up period. It is possible that such threats might be countered by early investment in group education, for example, helping the groups to identify potential sources of threat to the group’s safety from the very start and putting contingencies in place to support their address, for example, referring troublesome matters to the project co-ordinator for advice. Thus, group safety is a central consideration in the set-up of SHGs and crucial to a sense of belonging for progression of activities.

Capabilities amongst the membership and strong leadership appeared to be important to group operations.

Effective book-keeping and accounting enabled the active groups to plan and embark on income generation projects. This might possibly point to a recruitment strategy that purposively seeks to include sufficient caregivers with achievement at primary-level or even at secondary-level education, to ensure the smooth organisation of group business.

Alternatively, awareness of the relevance of capabilities and education to the success of a group might trigger early capacity-building amongst the membership, focusing on processes to support group management and organisation.

Finally, the active participation of caregivers in newly formed SHGs is subject to conditions both external and internal to the group. To sustain group development and to achieve growth in self-help activities, pathways for strategic support and capacity-building need to be in place at the start of the set-up. In such circumstances, the approach to SHGs has the potential to contribute to the evidence based on CBID/CBR initiatives development.
